# Is Post-ERCP Pancreatitis a Genetically Predisposed Complication?

**DOI:** 10.1155/2012/473960

**Published:** 2012-08-15

**Authors:** Konstantinos Mystakidis, George Kouklakis, Androniki Papoutsi, Vasilios D. Souftas, Eleni Efremidou, Dimitrios Kapetanos, Michail Pitiakoudis, Nikolaos Lyratzopoulos, Anastasios Karagiannakis, Alexandros Pantelios

**Affiliations:** ^1^Gastrointestinal Endoscopy Unit, Democritus University of Thrace, University General Hospital of Alexandroupolis, 68100 Dragana, Greece; ^2^Department of Medical Laboratories, Alexandreio Technological Institute of Thessaloniki, 57400 Thessaloniki, Greece; ^3^Department of Radiology, Democritus University of Thrace, University General Hospital of Alexandroupolis, 68100 Dragana, Greece; ^4^1st Surgical Department, Democritus University of Thrace, University General Hospital of Alexandroupolis, 68100 Dragana, Greece; ^5^Gastroenterology Department, George Papanikolaou Hospital, 57010 Thessaloniki, Greece

## Abstract

*Background/Objectives*. Pancreatitis remains the most common complication of ERCP. History of post-ERCP pancreatitis is an independent risk factor for a new episode, suggesting a genetic background. The N34S mutation in serine protease inhibitor Kazal type 1 (SPINK 1) gene may downregulate the threshold for the development of pancreatitis. The aim of the present study is to evaluate the presence of this mutation among patients with post-ERCP pancreatitis. *Methods*. During a period of four years, thirty patients with post-ERCP pancreatitis entered the study. Patients and procedural data were collected, focusing on risk factors for pancreatitis. Blood samples were taken for genetic testing for the presence of N34S mutation in SPINK 1 gene. After DNA extraction, we used an allele-specific polymerase chain reaction as an initial screening method for the N34S mutation, and in order to confirm the results and to determine the hetero- and homozygosity genotype status, we used a restriction fragment length polymorphism (RFLP) method. *Results*. None of the thirty patients was found to carry the N34S mutation, with both of the applied methods. Patients had an average of two of the known risk factors. *Conclusion*. SPINK1 N34S mutation does not seem to play a role in post-ERCP pancreatitis, but larger studies needed to confirm our results.

## 1. Introduction

Pancreatitis remains the most common complication of ERCP, with the reported incidence ranging from 2% to 9% [1]. Although 80% of cases are mild, a significant number of patients may develop severe pancreatitis, that means additional morbidity and risk for death. ERCP, despite the development of new diagnostic tools, remains a widely used procedure, so post-ERCP pancreatitis is a problem with significant impact.

 So far only the use of pancreatic stents in high risk patients has become a widely accepted practice to minimize the risk for post-ERCP pancreatitis [2]. However, this technique is costly and not widely available, so the question who are high risk patients remains vivid. 

 Several studies and meta-analyses helped us to recognize special factors that put an individual in high risk for the development of post-ERCP pancreatitis [3–10]. Among these factors special interest presents the history of post-ERCP pancreatitis as an independent risk factor for a new episode of post-ERCP pancreatitis. It seems that some individuals have a genetically predisposed susceptibility in this particular complication.

Genetic factors, such mutations in genes of cationic trypsinogen and CFTR, are known to play a causal role in the development of certain types of chronic pancreatitis [11]. Mutation in another gene, that codes the serine protease inhibitor Kazal type 1 (SPINK 1), has been found to act complementary to other genetic or environmental factors causing pancreatitis [12–14].

 The aim of the present study is to investigate the possible role of SPINK 1 gene mutations in the development of post-ERCP pancreatitis, examining the incidence of these mutations in this particular group of patients in comparison with the general population.

## 2. Patients and Methods

 For this purpose, a study was conducted in two high volume centers (more than 200 ERCPs/Y each). Between the years 2005 and 2008, the data of each “ERCP case” were collected according to a standard protocol. Patient data including demographics, past history and the indication for the procedure, were collected before the ERCP. Procedural data, including difficulty in cannulation (number of attempts on papilla), use of precut, pancreatic duct catheterization and opacification, findings of the examination, sphincterotomy, and all the other therapeutic maneuvers were recorded at the time of the procedure. All patients were followed up at least for 24 hours to monitor the development of complications after ERCP. Patients that developed post-ERCP pancreatitis, according to the widely accepted criteria (new onset of pancreatic-type abdominal pain, lasting at least for 24 hours, associated with a 3-fold increase in serum amylase in the same period) entered the study, after an inform consent was obtained. Exclusion criteria from the study were the presence of chronic pancreatitis and the refusal of consent. From the patients that entered, the study blood samples were collected for genetic analysis of SPINK1 gene. Patients were monitored during their hospitalization, and severity of pancreatitis was classified according to the consensus criteria [15].

## 3. Mutation Analysis

Genomic DNA was extracted from peripheral blood leucocytes according to established protocols using the Whole Blood DNA isolation Kit (Qiagen GmbH, Germany). Concentration of DNA solutions were determined by UV-Vis spectrophotometer. In order to identify N34S SPINK1 mutation as a possible risk factor for the post-ERCP pancreatitis, we applied the following approach. We used an allele-specific polymerase chain reaction as an initial screening method for the N34S mutation, according to a previously described report [16]. In order to confirm the results and to determine the hetero- and homozygosity genotype status, we used a restriction fragment length polymorphism (RFLP) method as previously described, with slight modifications [17].

### 3.1. Allele-Specific PCR

**Figure 1:**
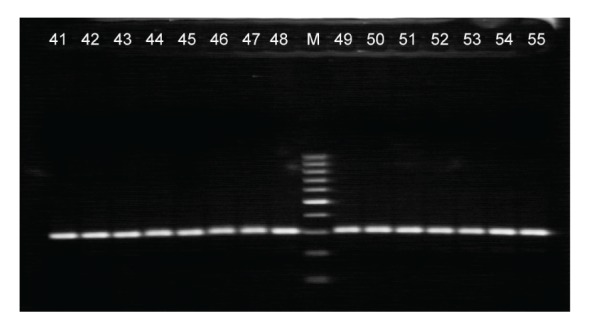
Allele-specific PCR analysis of the N34S mutation on exon 3 of the SPINK gene. The wild-type SPINK band is located at 285 bp. If the N34S mutation is present, a second band at 190 bp appears. The lanes 41 to 55 represent patients without the N34S mutation (normal). The lane marked with M represents the 100 bp DNA Ladder (Fermentas Life Sciences, Lithuania).

Briefly, 200 ng of genomic DNA were subtended to a 50 *μ*L reaction, containing 67 mM Tris-HCl (pH 8.8), 16 mM (NH_4_)_2_SO_4_, 0.01% Tween-20, 2 mM MgCl_2_, 250 *μ*M dNTPs, 3.0 U Taq DNA polymerase (Bioron International, Germany), and 100 ng of sense, antisense and mutation primers (see Table 1). The following cycling conditions were used for the above PCR reaction: an initial denaturation step at 94°C for five minutes, then 35 cycles at 94°C for 30 seconds, 59°C for 30 seconds, 72°C for 30 seconds, and a final elongation step at 72°C for five minutes. PCR products were electrophorized on a 2% agarose gel and stained with ethidium bromide for UV visualization. This allele-specific PCR generates two fragments (285 bp and 195 bp) when the N34S mutation is present, and one fragment of 285 bp for the wild-type genotype.  Figure 1 provide an overview of the results obtained with the allele-specific PCR.

### 3.2. RFLP Analysis

**Figure 2:**
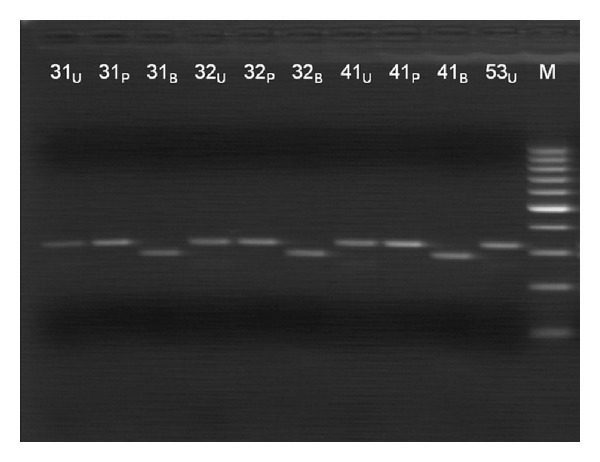
An overview of results obtained with N34S Restriction fragment length polymorphism analysis. Samples 31, 32, 41, and 53 are wild types. _U_undigested PCR product, _P_CR product digested with *Pst*I, _B_CR product digested with *Bsr*DI.

The primers we used for the RFLP analysis were designed (by Threadgold et al.) to amplify exon 3 of the SPINK1 gene, based on the published nucleotide sequence (GenBank, NM-003122). These primers (see Table 1) introduce a PstI endonuclease restriction site in sequences carrying the N34S variant, and a BsrDI endonuclease restriction site in wild-type sequences. Polymerase chain reaction was performed in a 50 *μ*L reaction volume, using 100 ng of genomic DNA template, 0.5 U Pfu DNA polymerase (Promega, Germany), 2 mM MgCl_2_, 200 *μ*M dNTPs (Fermentas Life Sciences, Lithuania), and 10 pmol of each primer. The cycling conditions we used for the RFLP method were as follows: an initial denaturation step at 94°C for 5 minutes, followed by 35 cycles at 94°C for 30 seconds, 60°C for 30 seconds, and 72°C for 1 minute. Then, the PCR products were digested with restriction endonucleases PstI and BsrDI. Twenty microliters of the PCR product was added to 10 U PstI (Fermentas Life Sciences, Lithuania), 1x digest buffer (Fermentas Life Sciences, Lithuania), and 1x bovine serum albumin (BSA) (Fermentas Life Sciences, Lithuania) to a final volume of 50 *μ*L and incubated at 37°C for one hour. The BsrDI digestion was performed in a 50 *μ*L reaction, consisting of 20 *μ*L PCR product, 10 U BsrDI (Fermentas Life Sciences, Lithuania), 1x digest buffer (Fermentas Life Sciences, Lithuania) and 1x bovine serum albumin (BSA) (Fermentas Life Sciences, Lithuania) and incubation at 55°C for one hour. Heat inactivation of the digestion reaction was performed for both digestions at 80°C for 15 minutes. The digestion products analysed by agarose gel electrophoresis using a 3% (w/v) precast agarose gels (Biorad Laboratories Inc., USA), 1X TAE buffer, and 0.5 mg/ml ethidium bromide. The undigested PCR products were 0320 bp in length. After digestion with PstI restriction endonuclease, a product of 286 bp obtained from mutant sequences. After digestion with BsrDI, a product of 286 bp obtained from wild-type sequences. Heterozygote samples produced both products of 320 bp and 286 bp after digestion with either endonuclease. To validate the RFLP analysis further, the BsrDI digestions was performed alternatively at 37°C overnight. The results obtained were identical in both experiments. An overview of results obtained with the RFLP methods are provided by Figure 2. 

## 4. Results

Between the years 2005 and 2008, 1162 patients underwent 1247 procedures, mainly for therapeutic purpose. Pancreatitis developed in 34 patients (2,7%). From these, 30 patients finally entered the study. From the remaining 4, one had chronic pancreatitis, and three refuted consent. From the 30 patients who entered the study, 21(70%) were women and 9 were men (30%), with a mean age of 63 years old (STDEV 13). Pancreatitis was classified, according to Cotton criteria [15], as mild in 17 patients (57%), moderate in 10 patients (33%), and severe in 3 patients (10%). We did not detect the N34S mutation among our study group with both methods of, allele-specific PCR and RLFP. Patients had an average of 2 risk factors/per pt. None of the patients in our group fulfilled the criteria of SOD, and we did not have patients with prior history of post-ERCP pancreatitis. The distribution of the remaining risk factors is presented in Table 2. The mean number of guidewire passes into the pancreatic duct was 2,47 (STDEV 3,46).

## 5. Discussion

SPINK 1 is a 6,5 KDa protein consisting of 79 amino acids, including a 23 amino acid signal peptide. It is synthesized in the pancreatic acinar cell and its central role is the protection of the pancreas from the prematurely activated trypsin. It has been estimated that SPINK 1 is capable of inhibiting up to 20% of trypsin within the pancreas, but SPINK/trypsin ratio likely varies depending on the state of inflammation in the gland. SPINK 1 protein is coded from a gene on the chromosome 5, that is approximately 7.5 Kb long, and contains four exons. The N34S mutation, caused by an c.101A > G transition in exon 3, was firstly reported by Chen et al. in 2000 [12]. However, the association between N34S mutation and pancreatitis became evident in two subsequent studies. Initially Witt et al. found a strong association of this mutation with “idiopathic” chronic pancreatitis, and they further speculated that these mutations cause the disease in an autosomal recessive manner [13]. However, Pfützer et al. in another major study suggested that these mutations, which are also present in about 2% of the healthy population, do not cause the disease by themselves, but rather modify the disease, possibly by lowering the threshold for pancreatitis from other genetic or environmental factors [14]. Several studies worldwide have confirmed the association between SPINK 1 mutations and idiopathic chronic pancreatitis, and as a consequence other groups investigate the role of these mutations in other types of chronic pancreatitis. So SPINK 1 mutations were found to be associated with tropical pancreatitis and to a lesser degree with alcoholic chronic pancreatitis [18–25]. Moreover, a recent study revealed that these mutations may play a role in recurrent episodes of acute pancreatitis [26]. 

 Post-ERCP pancreatitis is a type of acute pancreatitis that shares the same pathophysiological characteristics with any other type of acute pancreatitis. As we know, history of post-ERCP pancreatitis puts an individual in high risk for the development of a new episode of post-ERCP pancreatitis, independently from the other known factors. This implies that some individuals may have a genetic susceptibility in this complication. The N34S mutation, as mentioned earlier, may act complementary to another factor such as the irritation of the gland during ERCP for the development of pancreatitis. Lempinen et al. found that patients with post-ERCP pancreatitis had significantly higher levels of SPINK 1 from patients without pancreatitis, as a consequence of the induction of enzyme synthesis and secretion by the inflammation [27]. SPINK1 gene mutations may downregulate enzyme's protective role against the irritation that is caused to the gland by the ERCP maneuvers.

 Despite this logical approach, our study did not reveal this particular mutation among patients with post-ERCP pancreatitis. In contrast, patients had an average of two of the known risk factors. The limitation of the study is the small size, due to lack of many cases of post-ERCP pancreatitis. Moreover, data about the incidence of SPINK1 mutations in our country are lacking and maybe our ethnic origin represents a population with a low burden of these mutations. However, the fact that some persons are more susceptible than others, independently from the presence of other risk factors, strongly suggest the role of an unknown genetic factor. Large multicenter studies are needed to evaluate this hypothesis, examining the presence of this or other mutations in SPINK 1, or other relevant genes, in order to extract safer results.

 Conclusively according to our data, we have not got any clues that N34S SPINK 1 mutation plays a role in post-ERCP-pancreatitis, but further studies are needed to confirm our results. 

## Figures and Tables

**Table 1:** Sequences of oligonucleotide Primers of the SPINKI gene.

Primers	Allele-specific PCR	RFLP method
Sense	5′-CAATCACAGTTATTCCCCAGAG-3′	5′-TTCTGTTTAATTCCATTTTTAGGCCAAATGCTGCA-3′
Antisense	5′-GTTTGCTTTTCTCGGGGTGAG-3′	5′-GGCTTTTATCATACAAGTGACTTCT-3′
Mutation	5′-CCATTTTTAGGCCAAATGTTACAG-3′	

PCR: polymerase chain reaction; RFLP: restriction fragment length polymorphism.

**Table 2:** Risk factors for the development of post-ERCP pancreatitis.

Risk factors	Number of patients	Percentages (%)
Age <60 y	11	37
Female gender	21	70
History of pancreatitis	3	10
Difficult cannulation ≥5 attempts on papilla	5	17
Precut	3	10
Pancreatogram	18	60
